# Metabolic Profiles and cDNA-AFLP Analysis of *Salvia miltiorrhiza* and *Salvia castanea* Diel f. *tomentosa* Stib

**DOI:** 10.1371/journal.pone.0029678

**Published:** 2012-01-30

**Authors:** Dongfeng Yang, Pengda Ma, Xiao Liang, Zongsuo Liang, Meixiang Zhang, Shuang Shen, Hongyun Liu, Yan Liu

**Affiliations:** 1 College of Life Science, Northwest A&F University, Yangling, China; 2 College of Life Science, Zhejiang Sci-Tech University, Hangzhou, China; 3 Tianjin Tasly Modern TCM Resources Company, Limited, Tianjin, People's Republic of China; University of California, United States of America

## Abstract

Plants of the genus *Salvia* produce various types of phenolic compounds and tanshinones which are effective for treatment of coronary heart disease. *Salvia miltiorrhiza* and *S. castanea* Diels f. *tomentosa* Stib are two important members of the genus. In this study, metabolic profiles and cDNA-AFLP analysis of four samples were employed to identify novel genes potentially involved in phenolic compounds and tanshinones biosynthesis, including the red roots from the two species and two tanshinone-free roots from *S. miltiorrhiza*. The results showed that the red roots of *S. castanea* Diels f. *tomentosa* Stib produced high contents of rosmarinic acid (21.77 mg/g) and tanshinone IIA (12.60 mg/g), but low content of salvianolic acid B (1.45 mg/g). The red roots of *S. miltiorrhiza* produced high content of salvianolic acid B (18.69 mg/g), while tanshinones accumulation in this sample was much less than that in *S. castanea* Diels f. *tomentosa* Stib. Tanshinones were not detected in the two tanshinone-free samples, which produced high contents of phenolic compounds. A cDNA-AFLP analysis with 128 primer pairs revealed that 2300 transcript derived fragments (TDFs) were differentially expressed among the four samples. About 323 TDFs were sequenced, of which 78 TDFs were annotated with known functions through BLASTX searching the Genbank database and 14 annotated TDFs were assigned into secondary metabolic pathways through searching the KEGGPATHWAY database. The quantitative real-time PCR analysis indicated that the expression of 9 TDFs was positively correlated with accumulation of phenolic compounds and tanshinones. These TDFs additionally showed coordinated transcriptional response with 6 previously-identified genes involved in biosynthesis of tanshinones and phenolic compounds in *S. miltiorrhiza* hairy roots treated with yeast extract. The sequence data in the present work not only provided us candidate genes involved in phenolic compounds and tanshinones biosynthesis but also gave us further insight into secondary metabolism in *Salvia*.

## Introduction

Secondary metabolites, which have been used by human for thousands of years, are an important research field in crop breeding and metabolic engineering. Due to enormous economic interests, the investigation of secondary metabolism has never been stopped since the introduction of tracer technology [1]. In recent years, the combination of metabolic profile and transcriptome has been widely used for discovery of secondary metabolism-related genes in plants [2], [3]. cDNA-amplified fragment length polymorphism (cDNA-AFLP) is one of the most robust and sensitive transcriptomic technologies for gene discovery [4] and offers an attractive method to identify genes involved in secondary metabolism of non-model plants [5]. By combining targeted metabolite analysis and cDNA-AFLP based transcript profiling of tobacco BY-2 [5] and *Catharanthus roseus* cells [6], many novel genes were identified and some of them were potentially involved in secondary metabolism. Ziegler et al. used cDNA-AFLP and macroarray analysis to compare the benzylisoquinoline biosynthesis in morphine-containing *Papaver somniferum* and eight morphine-free *Papaver* species, and then an *O*-methyltransferase was identified [2].

**Figure 1:**
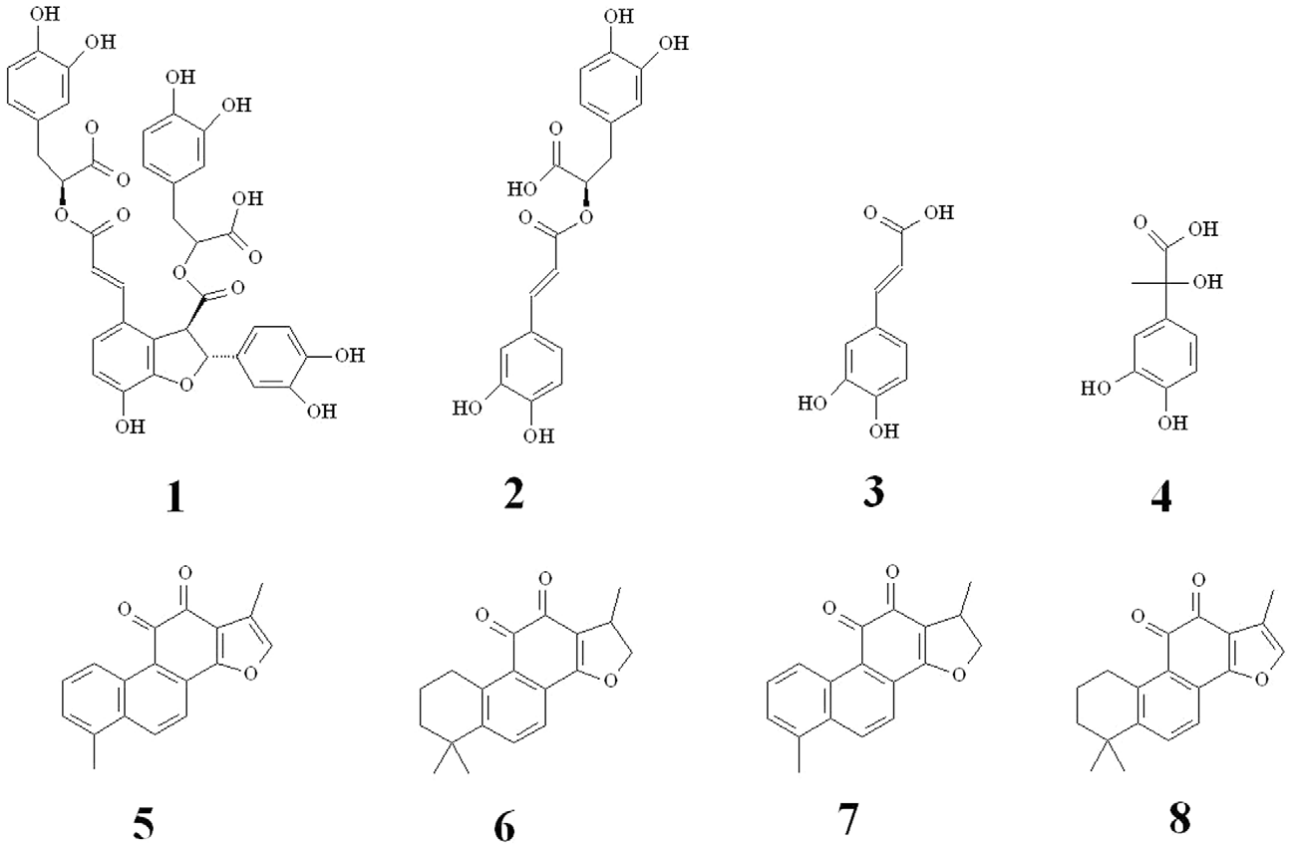
Chemical structures of four phenolic compounds and four tanshinones. (1) salvianolic acid B, (2) rosmarinic acid, (3) caffeic acid, (4) danshensu, (5) dihydrotanshinone I, (6) cryptotanshinone, (7) tanshinone I, (8) tanshinone IIA.

There are about 40 *Salvia* species in China. *Salvia miltiorrhiza* (Danshen in Chinese) is one of the most important and popular traditional Chinese medicinal plants, and has been widely used in prevention and treatment of coronary heart disease, chronic renal failure, atherosclerosis, myocardial infarction and cirrhosis [7]. *S. miltiorrhiza* was proposed as an appropriate potential model plant species for research in traditional Chinese medicine because of its medicinal purposes and biological characteristics [8]. There are two major groups of active ingredients in *S. miltiorrhiza* roots, tanshinones and phenolic compounds (Figure 1). The former, mainly including tanshinone IIA, cryptotanshinone, tanshinone I and dihydrotanshinone I, are biosynthesized through the mevalonate (MVA) and the 2-C-methyl-D-erythritol-4-phosphate (MEP) pathways [9]. The latter, mainly including salvianolic acid B, rosmarinic acid, caffeic acid and danshensu, are biosynthesized via the phenylpropanoid and the tyrosine-derived pathways [10]. These active ingredients show a variety of biological activities such as blood circulation improvement, antioxidant and myocardial infarction prevention [7]. Many studies have attempted to elucidate the biosynthesis of tanshinones and phenolic compounds in *S. miltiorrhiza*. A microarray chip from the cDNA library of *S. miltiorrhiza* roots was manufactured to identify novel genes participating in tanshinones biosynthesis [11]. A substantial EST dataset for *S. miltiorrhiza* root was generated on the Roche 454-GS FLX Titanium platform and some putative genes involved in tanshinones and phenolic compounds biosynthesis were obtained [12]. Using *de novo* transcriptome sequencing in *S. miltiorrhiza*, Hua et al. identified a set of putative genes involved in pathways of secondary metabolism [13]. Until now, several genes involved in tanshinones and phenolic compounds biosynthesis have been cloned, such as genes encoding 3-hydroxy-3-methylglutaryl coenzyme A reductase (HMGR) [14], 1-deoxy-D-xylulose 5-phosphate reductoisomerase (DXR) [15], [16], *ent*-kaurene synthase (KS) [11], phenylalanine ammonia-lyase (PAL) [17] and tyrosine aminotransferase (TAT) [18]. However our knowledge about secondary metabolism in *S. miltiorrhiza* is far from complete and most of genes in the terminal steps of tanshinones and phenolic compounds biosynthesis are still unknown.

*S. castanea* Diels f. *tomentosa* Stib mainly produced in Linzhi of Tibet is a forma of *S. castanea* Diels and grows at the altitude between 2500 and 3750 meters [19]. *S. castanea* Diels f. *tomentosa* Stib has the similar therapeutic effects as *S. miltiorrhiza*. Our previous work has revealed that tanshinones content in *S. castanea* Diels f. *tomentosa* Stib was higher than that in *S. miltiorrhiza*. However, salvianolic acid B content in *S. castanea* Diels f. *tomentosa* Stib was much less than that in *S. miltiorrhiza* [20]. In addition, two tanshinone-free root samples of *S. miltiorrhiza* were obtained in our lab. One was the hydroponic root and another was the white root of *S. miltiorrhiza*. The differences of secondary metabolites accumulation in these samples indicated that genes involved in biosynthesis of tanshinones and phenolic compounds were probable differentially expressed. In this study, metabolic profiles and cDNA-AFLP analysis of the hydroponic, white and red roots of *S. miltiorrhiza*, and the red roots of *S. castanea* Diels f. *tomentosa* Stib were performed to identify novel genes involved in tanshinones and phenolic compounds biosynthesis. The interesting fragments involved in secondary metabolism were validated further by analysis of gene expression and secondary metabolites accumulation in *S. miltiorrhiza* hairy roots.

## Results and Discussion

### Metabolic profiles of *S. miltiorrhiza* and *S. castanea* Diels f. *tomentosa* Stib

**Figure 2:**
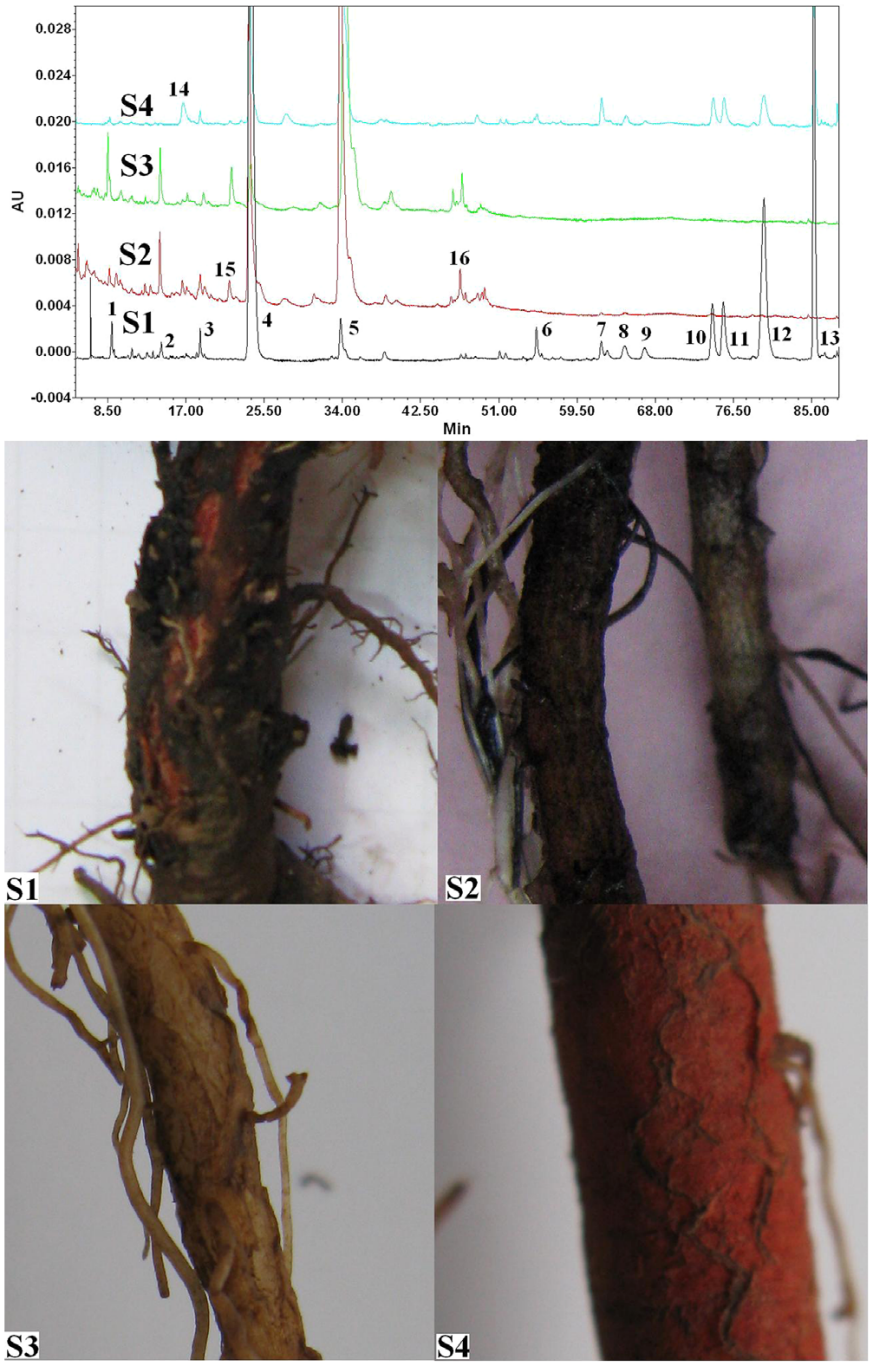
Metabolic profiles of the red roots of *S. castanea* Diels f. *tomentosa* Stib (S1), and the hydroponic (S2), white (S3) and red (S4) roots of *S. miltiorrhiza* by HPLC at 280 nm. Peak 1, danshensu; peak 2, caffeic acid; peak 4, rosmarinic acid; peak 5, salvianolic acid B; peak 7, dihydrotanshinone I; peak 10, cryptotanshinone; peak 11, tanshinone I; peak 13 tanshinone IIA.

With an appropriate HPLC method, metabolic profiles of the four samples (S1, S2, S3 and S4) were investigated. Contents of 8 active ingredients in the samples were determined including danshensu, caffeic acid, rosmarinic acid, salvianolic acid B, tanshinone IIA, cryptotanshinone, dihydrotanshinone I and tanshinone I. The results were means ± standard deviation (S.D.) of three biological replications (Figure 2 and Table 1). As a result, 13 major peaks were detected in S1. Rosmarinic acid (peak 4) was the main phenolic compound (21.77 mg/g, 36% of the total peak area). Contents of danshensu (peak 1), caffeic acid (peak 2) and salvianolic acid B (peak 5) in S1 were 0.91, 0.12 and 1.45 mg/g, respectively. Tanshinone IIA (peak 13) was the main tanshinone (12.6 mg/g, 26% of the total peak area). Contents of dihydrotanshinone I (peak 7), cryptotanshinone (peak 10) and tanshinone I (peak 11) in S1 were 0.28, 1.7 and 2.96 mg/g, respectively. The 13 peaks were also detected in S4. However, their peak areas in S4 were much less than those in S1 except peak 5 and 7. Salvianolic acid B was the main phenolic compound in S4 (18.69 mg/g) and its content was 12.9-fold of that in S1. Content of rosmarinic acid in S4 was just 2.27 mg/g (less than 11% of that in S1). Tanshinone IIA was the main tanshinone in S4 (2.66 mg/g, 20% of that in S1). Contents of tanshinone I, cryptotanshinone, dihydrotanshinone I in S4 were 0.72, 0.40, and 0.97 mg/g, respectively. Additionally, peak 14 was only detected in S4. Metabolite profiles of S1 and S4 in this study coincided with our earlier reports [20]. Tanshinones were not detected in S2 and S3. However, high contents of phenolic compounds in S2 and S3 were observed. Peak 15 and 16 were detected only in S2 and S3. Salvianolic acid B (51.40 mg/g) and rosmarinic acid (25.58 mg/g) were the main phenolic compounds in S2, while salvianolic acid B (24.16 mg/g) was the main ingredient in S3. Content of rosmarinic acid in S3 was very low.

**Table 1:** Contents of active components in different samples (mg/g) (*n = 3*).

Component	S1	S2	S3	S4
Danshensu	0.91±0.12	0.34±0.02	1.85±0.14	0.16±0.01
Caffeic acid	0.12±0.02	0.58±0.02	0.53±0.02	-
Rosmarinic acid	21.77±1.42	25.58±1.35	1.26±0.01	2.27±0.18
Salvianolic acid B	1.45±0.17	51.40±2.41	24.16±1.36	18.69±0.12
Dihydrotanshinone I	0.28±0.01	-	-	0.72±0.04
Cryptotanshinone	1.70±0.01	-	-	0.40±0.02
Tanshinone I	2.96±0.11	-	-	0.97±0.03
Tanshinone IIA	12.60±0.83	-	-	2.66±0.15

S1: the red roots of *S. castanea* Diels f. *tomentosa* Stib; S2: the hydroponic roots of *S. miltiorrhiza*; S3: the white roots of *S. miltiorrhiza*; S4: the red roots of *S. miltiorrhiza*. Three biological replications of each sample were performed. The results were represented by their means ± standard deviation (S.D.).

The gene discovery based on the difference of secondary metabolite accumulation has been widely reported [2], [3]. By analysis of morphine-containing *Papaver somniferum* and eight morphine-free *Papaver* species, an *O*-methyltransferase involved in benzylisoquinoline biosynthesis was discovered [2]. Using rose flowers from tetraploid scented and nonscented cultivars, several novel flower scent-related candidate genes were identified [3]. In this study, the difference of phenolic compounds and tanshinones accumulation in the hydroponic, white and red roots of *S. miltiorrhiza* and the red roots of *S. castanea* Diels f. *tomentosa* Stib indicated that genes involved in secondary metabolism were probably differentially expressed in these samples.

### Isolation of differentially expressed genes

RNA isolation is an essential step to study gene expression at the mRNA level. However, RNA extraction from roots of *S. miltiorrhiza* is difficult because of large amounts of polysaccharides, polyphenole and secondary metabolites. In this study, the CTAB-Li method was selected as the suitable method for RNA extraction from roots of *Salvia* on the base of integrity and purity of RNA. To select a suitable restriction enzyme combination for cDNA-AFLP analysis of *S. miltiorrhiza*, several enzyme combinations were tested and the combination of *Bst*YI and *Mse*I produced an acceptable range of fragment sizes.

A total of 128 primer combinations were used to selectively amplify the expressed genes. Differentially expressed transcript derived fragments (TDFs) were extracted from the gel and used as templates for re-amplification by PCR. The cDNA-AFLP fragments were highly reproducible as the band intensities were similar from the three biological replications. All the visible TDFs between 100 and 700 bp were counted. As a result, 2300 TDFs based on presence or absence between S4 and the other three samples were differentially expressed in the four samples. Of 928 differentially expressed TDFs between S2 and S4, 391 were present in S4 and 537 present in S2. Of 596 differentially expressed TDFs between S3 and S4, 217 were present in S3 and 379 present in S4. Of 776 differentially expressed TDFs between S1 and S4, 385 were present in S3 and 291 present in S4. A total of 975 TDFs were isolated from the gel and re-amplified, and 573 of them were selected to sequence. Finally, 323 TDFs were successfully sequenced.

### Gene sequence analysis

The annotation approach was based on sequence similarity searches in database. The 323 TDFs were subjected to a BLASTX search against the NCBI non-rebundant protein database. The results showed that 109 (34%) TDFs had significant sequence similarities to protein (eValue≤10^−5^) and the remaining 214 (66%) failed to match in the database. It was indicated that the information about the genomes or transcriptomes of the two species was limited. Of the 109 TDFs, 15% were homologous to *vitis vinifera*, 9% homologous to *Ricinus communis* and 8% homologous to *Arabidopsis lyrate*.

GO assignments describe gene products in terms of their associated molecular functions, biological processes and cellular components. Blast2GO (B2G) is a bioinformatic tool for GO-based DNA or protein sequence annotation. The 109 TDFs were annotated by Blast2GO tool and 78 of them were successfully annotated (Table S1). About 60% of them were involved in biological processes and encoded a broad set of transcripts represented within cellular metabolic, primary metabolic and biosynthetic processes. A total of 67 TDFs were annotated within molecular function strategy and most of them were within oxidoreductase activity, protein binding and ATP binding. About 60% of the TDFs represented the strategy of cellular component, including plastid, chloroplast and cytoplasmic membrane-bounding vesicle and about 26% of them were in plastid. Due to the MEP pathway responsible for tanshinones biosynthesis was in plastid, these sequences within plastid might help us discover novel genes involved in tanshinones biosynthesis.

KEGG provides a reference knowledge base for linking genomes to life through the process of PATHWAY mapping. In this study, the 78 annotated TDFs were blasted against the KEGG database. As a result, 27 of them were highly homologous to the enzyme and 22 of them were assigned into the metabolic pathways in the database (Table S2). Excitingly, 14 TDFs were assigned into secondary metabolic pathways and mainly involved in biosynthesis of phenylpropanoids, alkaloids, terpenoids and steroids.

### Metabolic profiles and expression of key genes in YEL-treated *S. miltiorrhiza* hairy roots

Tanshinones, a group of diterpenoids are synthesized via the MVA and the MEP pathways. HMGR is the rate-limiting enzyme in the MVA pathway, DXR is the key enzyme in the MEP pathway and KS is also involved in tanshinones biosynthesis [11]. Phenolic compounds in *S. miltiorrhiza* are biosynthesized through the phenylpropanoid and the tyrosine-derived pathways [10]. PAL is a limiting enzyme in the phenylpropanoid pathway [27] and TAT is the first enzyme in the tyrosine-derived pathway [18]. The involvement of the two pathways in rosmarinic acid biosynthesis has been proven and RAS is a key enzyme [28]. In this study, contents of tanshinones and phenolic compounds as well as expression levels of *HMGR*, *DXR*, *KS*, *PAL*, *TAT* and *RAS* in *S. miltiorrhiza* hairy roots treated by YEL were investigated.

**Figure 3:**
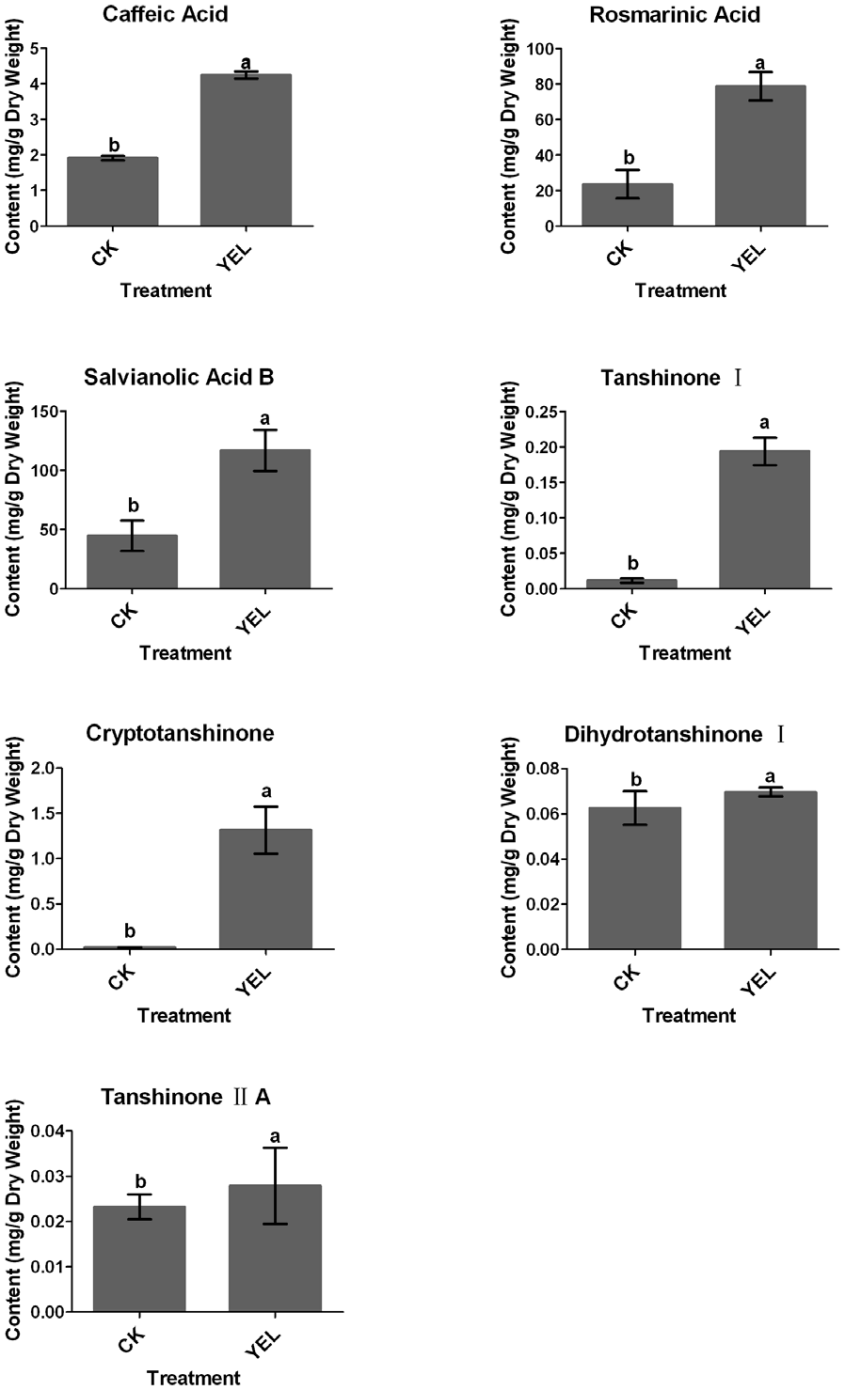
Accumulation of phenolic compounds and tanshinones in *S. miltiorrhiza* hairy roots induced by yeast elicitor. CK, the control; YEL, yeast extract liquid. Three biological replications were performed. The results were represented by their means ± standard deviation (S.D.). Different letters indicated significant difference at *p*≤0.05 using Duncan's multiple range test.

**Figure 4:**
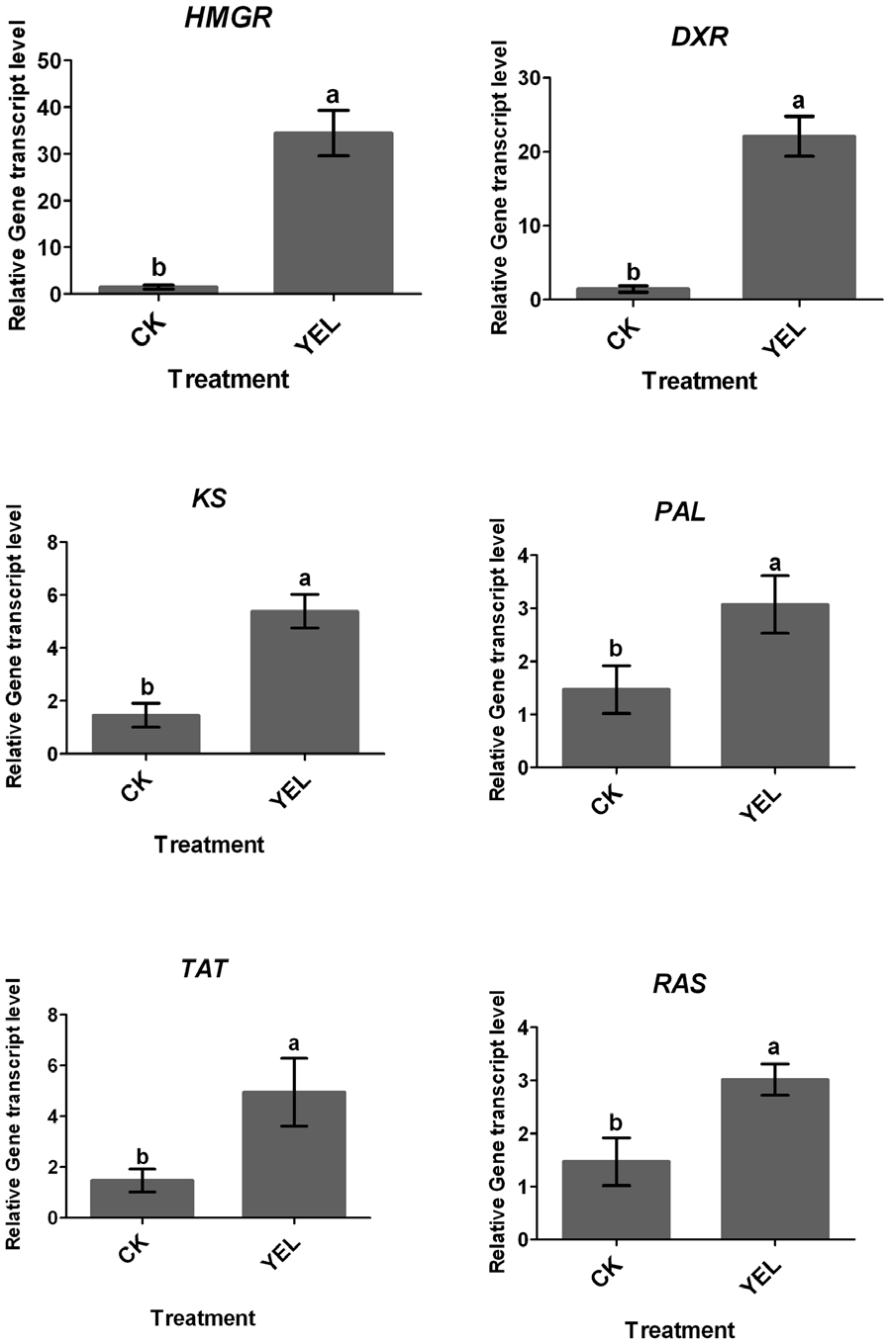
The relative expression levels of six genes involved in phenolic compounds and tanshinones biosynthesis in *S. miltiorrhiza* hairy roots induced by yeast elicitor. CK, the control; YEL, yeast extract liquid. HMGR, 3-hydroxy-3-methylglutaryl coenzyme A reductase; DXR, 1-deoxy-D-xylulose 5-phosphate reductoisomerase; KS, *ent*-kaurene synthase; PAL, phenylalanine ammonia-lyase; RAS, rosmarinic acid synthase; TAT, tyrosine aminotransferase. The relative expression levels of six genes in *S. miltiorrhiza* hairy roots induced by yeast elicitor were calculated as fold of those in the non treated hairy roots using the 2^−ΔΔCT^ method. All data were normalized to the housekeeping gene. Three biological replications were performed. The results were represented by their means ± standard deviation (S.D.). Different letters indicated significant difference at *p*≤0.05 using Duncan's multiple range test.

Both phenolic compounds and tanshinones were significantly stimulated by application of YEL (Figure 3). Contents of caffeic acid, rosmarinic acid and salvianolic acid B in YEL-treated hairy roots were increased by 1.2, 2.3 and 1.6-fold over the control levels, respectively. Correspondingly, gene expression involved in phenolic compounds biosynthesis was up-regulated by YEL (Figure 4). The mRNA levels of PAL, TAT and RAS were increased by 3.2, 6.6 and 2.6-fold over the control levels, respectively. The involvement of PAL in rosmarinic acid biosynthesis has been confirmed in suspension cultures of *Coleus blume* [29]. However, Yan et al. reported that YEL-induced rosmarinic acid production in *S. miltiorrhiza* hairy roots was correlated with TAT but not PAL activity [30]. Whereas, we observed that PAL might be the key enzyme for the biosynthesis of salvianolic acid B and caffeic acid in the *S. miltiorrhiza* cell culture [31]. In present work, we observed that *TAT* expression was more sensitive to YEL treatment than *PAL* expression.

Contents of tanshinone I and cryptotanshinone in YEL-treated *S. miltiorrhiza* hairy roots were increased by 16.8 and 73.1-fold over the control levels, while dihydrotanshinone I and tanshinone IIA accumulation were almost unaffected by YEL. Expression of *HMGR*, *DXR* and *KS* involved in tanshinones biosynthesis was up-regulated by YEL treatment and their expression levels were increased by 30.4, 17.4 and 3.7-fold over the control levels, respectively. *HMGR* and *DXR* expression was more sensitive to YEL elicitor than *KS* expression. It has been reported that *KS* expression was significantly induced by methyl jasmonate (MJ) and was probably involved in tanshinones biosynthesis [11]. The similar results were observed in this study. These results indicated that YEL was an effective elicitor to induce tanshinones and phenolic compounds accumulation. Correspondingly, expression of tanshinones and phenolic compounds biosynthesis-related genes was up-regulated by YEL treatment.

### Quantitative RT-PCR analysis of YE-induced differentially expressed TDFs in *S. miltiorrhiza* hairy roots

Co-expression analysis, which is based on the premise that a set of genes involved in a biological process are co-regulated or co-expressed under given conditions, has been successfully used to identify novel genes for secondary metabolism. If an unknown gene is co-expressed with known genes in a metabolic pathway, the unknown gene is probably involved in the pathway [32], [33]. Through this approach, many genes involved in secondary metabolism of *Arabidopsis thaliana* have been identified [32], [33]. To verify the correlation between expression of differentially expressed TDFs and accumulation of secondary metabolites in *S. miltiorrhiza* hairy roots, quantitative RT-PCR analysis was carried out for 16 TDFs including C841 and O641 (oxidation reduction); O741, O743 and G841 (organic acid metabolic process); E844 and G741 (regulation of transcription); G843, I842, L341, M442, P541, N441, A1410, A1418 and A346 (other interesting genes). These TDFs were just present in S4 but not in the other three samples.

As shown in Table 2, expression of two secreted protein genes (A346 and I842) was down-regulated by YEL. N441, O743 and P541 were annotated as lectin, glyceraldehyde-3-phosphate dehydrogenase and transcription factor, and their expression remained constant under YEL treatment. It was indicated that these genes were probably not involved in tanshinones and phenolic compounds biosynthesis.

**Table 2:** The relative expression levels of 16 TDFs in *S. miltiorrhiza* hairy roots induced by yeast elicitor (*n = 3*).

Fragment	Relative transcript level(fold of the control)	Fragment	Relative transcript level(fold of the control)
A148	72.97±14.32	I842	0.21±0.04
A1410	29.82±4.90	L341	3.26±0.67
A346	0.09±0.06	M442	2.76±0.56
C841	2.16±0.39	N441	0.95±0.26
E844	2.78±0.67	O641	1.94±0.29
G741	3.49±0.54	O741	1.96±0.23
G841	2.65±0.69	O743	1.09±0.39
G843	5.94±0.60	P541	1.01±0.24

The relative expression levels of 16 TDFs in *S. miltiorrhiza* hairy roots induced by yeast elicitor were calculated as fold of those in the non treated hairy roots using the 2^−ΔΔCT^ method. All data were normalized to the housekeeping gene. Three biological replications were performed. The results were represented by their means ± standard deviation (S.D.).

Jasmonic acid and its conjugates, methyl jasmonate (MJ) collectively referred to as jasmonates, are small signaling molecules. The potency of jasmonates to elicit secondary metabolism in cell cultures has been revealed [34]. Lipoxygenase (LOX) catalyzing the oxidation of polyunsaturated fatty acids was a key gene involved in jasmonates biosynthesis [35]. Increasing evidences showed that lipoxygenase participated in the biosynthesis of secondary metabolites [36]. The mechanism of transcriptional regulation by MJ was largely unknown until the recent discovery of a novel family of transcriptional regulators called jasmonate zim domain (JAZ) protein, which was highly induced by MJ treatment [37]. In *S. miltiorrhiza*, the previous work has shown that MJ elicits the production of tanshinones and phenolic compounds [10], [38]. In this study, TDFs G741 and G841 showed significant sequence similarity to a JAZ protein and lipoxygenase, respectively. Their expression levels were increased by 1.7 and 1.3-fold over the control levels by YEL. It suggested that jasmonates were probably involved in tanshinones and phenolic compounds biosynthesis. The up-regulation of C841, a catalase homologous gene, indicated that the burst of reactive oxygen species (ROS) triggered by YEL was probably involved in tanshinones and phenolic compounds biosynthesis.

Pyruvate was precursor of the MEP pathway and a pyruvate decarboxylase catalyzed formation of terpenoid ketones [39]. Carotenoid was biosynthesized from the MEP pathway and an ATP citrate synthase was involved in carotenoid biosynthesis [40]. In this study, M442 and O741 were pyruvate decarboxylase and ATP citrate synthase, respectively. By YEL treatment, up-regulation of M442 and O741 indicated that they might be involved in tanshinones biosynthesis. E844 and G843 were two genes encoding HD domain class transcription factor and dihydroflavonol reductase which were involved in biosynthesis of anthocyanin derived from the phenylpropanoid pathway [41], [42]. The up-regulation E844 and G843 indicated that they were probably involved in phenolic compounds biosynthesis.

L341, A148 and A1410 were the unknown genes. Noticeably, their expression levels were increased by 3.3, 29.8 and 73.0-fold over the control levels, respectively. Probably, they were the novel genes involved in phenolic compounds and tanshinones biosynthesis.

In conclusion, we revealed that both *S. miltiorrhiza* and *S. castanea* Diel f. *tomentosa* Stib could produce large amounts of tanshinones and phenolic compounds. Tanshinone IIA and rosmarinic acid contents in *S. castanea* Diel f. *tomentosa* Stib were much higher than those in *S. miltiorrhiza*, while salvianolic acid B content in *S. castanea* Diel f. *tomentosa* Stib were only 8% of that in *S. miltiorrhiza*. The white roots and the hydroponic roots of *S. miltiorrhiza* were two tanshinone-free and phenolic compounds-containing samples. About 2300 differentially expressed TDFs were generated by cDNA-AFLP analysis of the four samples. Of 323 TDFs successfully sequenced, 78 TDFs were annotated with known function. At least 14 annotated TDFs were assigned into secondary metabolic pathways through searching the KEGGPATHWAY database and they were mainly involved in biosynthesis of phenylpropanoids, alkaloids, terpenoids and steroids. The expression levels of 9 TDFs (only present in the red roots of *S. miltiorrhiza*) were positively related to tanshinones and phenolic compounds production and were also co-regulated with phenolic compounds and tanshinones biosynthesis-related genes by YEL. They were genes encoding lipoxygenase, jasmonate zim-domain protein, pyruvate decarboxylase, catalase, cinnamyl alcohol dehydrogenase-like protein, HD domain class transcription factor, dihydroflavonol reductase and two unknown genes. The sequence data in the present work not only provided us candidate genes involved in biosynthesis of tanshinones and phenolic compounds but also gave us further insight into secondary metabolism in *Salvia*.

## Materials and Methods

### Plant materials

The red roots of *S. castanea* Diels f. *tomentosa* Stib (S1) and *S. miltiorrhiza* (S4) and the white roots of *S. miltiorrhiza* (S3) growing for three month in the wild were obtained from the medical plants garden of Northwest A&F University in Shaanxi province (June 10, 2009). *S. miltiorrhiza* seedlings were cultured in MS liquid medium under natural temperature and photoperiod for 120 days, and then the hydroponic roots (S2) were harvested. Three different plants each for S1, S2, S3, and S4 were collected for analysis of metabolic profiles and cDNA-AFLP. The plants were authenticated by Professor Yuejin Zhang of Northwest A&F University. The root samples were frozen in liquid nitrogen immediately, and then stored at −80°C until RNA isolation.

### Hairy root culture and treatment

*S. miltiorrhiza* hairy roots were derived after the infection of plantlets with *Agrobacterium rhizogenes* bacterium (ATCC15834). Experiments in this study were carried out in a 250 mL shake-flask on an orbital shaker running at 110–120 rpm and at 25°C in darkness [21]. Each flask was filled with 50 mL liquid medium and inoculated with 0.3 g fresh hairy roots from a 3-week-old shake-flask culture. The liquid medium was made of hormone-free MS medium with 30 g/L sucrose but without ammonium nitrate as previously described [21].

The polysaccharide fraction of yeast extract was prepared by ethanol precipitation as described [22]. The treatment with yeast elicitor liquid (YEL) (containing 100 µg/mL polysaccharide) were conducted on day 18 post inoculation of the hairy root culture. The equal volume of distilled water was added to the hairy root culture as the control. Three independent biological replications were performed.

### HPLC analysis of tanshinones and phenolic compounds

HPLC analysis was performed using a Waters (Milford, MA, USA) system with a binary pump and Photodiode Array Detector (DAD) as previously described [23]. A SunFire C18 column (250 mm×4.6 mm, 5 µm) was used. Data were acquired and processed using Empower 2 software.

Root samples collected from S1, S2, S3, S4 and *S. miltiorrhiza* hairy root culture were blotted dry with paper towels and dried at 45°C in an oven until constant weight. The dried roots were ground into a fine powder in a mortar with a pestle and sieved through a 0.45-mm screen. Each sample (0.1 g) was extracted ultrasonically with 2 mL of methanol-water solution (7∶3, v/v) for 45 min, the extract was centrifuged at 10,000 rpm for 15 min, and then the supernatant was filtered through a 0.45-µm filter. Separation was achieved by a gradient elution with acetonitrile and water (1% H_3_PO_4_). The effluent was monitored between 200 and 400 nm by DAD. Three biological replicates of each sample were analyzed. The results were represented by means ±S.D. of three replicates.

### RNA isolation, cDNA synthesis and cDNA-AFLP analysis

Root samples collecting from S1, S2, S3 and S4 were frozen in liquid nitrogen and strored at −80°C. Total RNA was isolated from about 0.2 g of each frozen sample by CTAB-Li method according the literature [24]. RNA purity and integrity were determined by running 2 µL of total RNA in a formamide denaturing gel along with an RNA ladder (Taraka, Japan). Genomic DNA in RNA preparation was removed by DNase I (Takara, Japan).

The cDNA synthesis and AFLP analysis was performed as described by the protocol [25]. Briefly, the first-strand cDNA was synthesized by SuperScript™ III Reverse Transcriptase (Invitrogen, USA) with an oligo dT_20_ primer according to the manufacture's instruction. The second-strand cDNA synthesis was performed by strand displacement with *Escherichia coli* ligase (Takara, Japan), DNA polymerase I (Takara, Japan) and RNase H (NEB, USA). The reaction mixture was incubated for 1 h at 12°C and for another 1 h at 22°C. The purified cDNA template was digested with restriction enzyme *Bst*YI (NEB, USA) for 2 h at 60°C and with *Mse*I (NEB, USA) for another 2 h at 37°C. The digested products were ligated by T4 DNA ligase (Takara, Japan) with adapters complementary to the restriction site of *Bst*YI (5′-CTCGTAGACTGCGTAGT-3′ and 5′-GATCACTACGCAGTCTAC-3′) and *Mse*I (5′-GACGATGAGTCCTGAG-3′ and 5′-TACTCAGGACTCAT-3′) for 3 h at 37°C. The ligated fragments were pre-amplified using *Mse*I primer (5′-GATGAGTCCTGAGTAA-3′) and *Bst*YI primer (5′-GACTGCGTAGTGATC(T/C)-3′) for 25 cycles (94°C for 30 s, 56°C for 1 min and 72°C for 1 min). The pre-amplified fragments were diluted 600-fold and 5 µL of aliquot was selectively amplified using 128 primer combinations (*Bst*YI primer 5′-GACTGCGTAGTGATC(T/C)N-3′ and *Mse*I primer 5′-GATGAGTCCTGAGTAANN-3′, where N represented the selective nucleotide). The amplification was performed using a touchdown amplification program (94°C for 3 min; 13 cycles of 94°C for 30 s, 65°C for 30 s (reducing 0.7°C per cycle) and 72°C for 1.0 min; 23 cycles of 94°C for 30 s, 56°C for 30 s and 72°C for 1.0 min; 72°C for 10 min). Selective amplification products were separated on 6% denaturing polyacrylamide sequencing gel with 0.5× TBE electrophoresis buffer (0.089 M Tris-borate, 0.089 M boric acid and 0.002 M EDTA). Images of TDFs were developed by silver staining.

### Characterization of AFLP fragments

Selective amplification products from 3 biological replicates of S1, S2, S3 and S4 were loaded and run for 2 h in a 6% denaturing polyacrylamide sequencing gel. Bands corresponding to differentially expressed genes of interest based on presence or absence between S4 and the other three samples were cut from the gel with a sharp razor blade, with maximum care to avoid any contaminating fragments. Each cut fragment was resolved in 30 µL 1× TE buffer for 60 min at room temperature and centrifuged at 18,000 rpm for 20 min at 4°C. Three microliters of the aliquot were used as a template for re-amplification using the pre-amplification primers and the following PCR program: 13 cycles of 94°C for 30 s, 65°C for 30 s (reducing 0.7°C per cycle) and 72°C for 1.0 min; 23 cycles of 94°C for 30 s, 56°C for 30 s and 72°C for 1.0 min. The re-amplification product was checked on a 1% agarose gel. A single clear band was indicative of a single amplified fragment. The obtained re-amplified fragments were purified and sequenced directly.

The sequences were compared to the GenBank database by BLASTX sequence alignments (E-value≤1×10^−5^; http://www.ncbi.nlm.nih.gov/BLAST) and Gene Ontology (GO) annotation was performed by BLAST2GO tool software v2.4.8. (http://www.blast2go.org/start_blast2go). Sequences which found homology with annotated sequences were annotated according to GO terms. Finally, the sequences were mapped to metabolic pathways using Kyoto Encyclopedia of Genes and Genomes (KEGG, http://www.genome.jp/kegg/) through homology searches.

### Quantitative real-time PCR analysis

*S. miltiorrhiza* hairy roots 24 h after YEL treatment was homogenized in liquid nitrogen into a fine powder. The total RNA was extracted by RNAiso™ Plus (Takara,Tokyo, Japan) and the cDNA was synthesized from 500 ng total RNA using PrimeScript® RT reagent Kit (Takara, Japan) according to the manufacturer's protocol. Primers used for cDNA synthesis were oligo dT primer and random 6 mers supplied in the Kit. The housekeeping gene (18S rRNA gene) was used as an internal control to normalize for variation in the amount of cDNA template. The quantitative RT-PCR was performed to detect the expression of *HMGR*, *DXR*, *KS*, *PAL*, *TAT*, *RAS* and 16 TDFs genes by a Bio-Rad CFX96 system (Bio-Rad, USA) with Brilliant II SYBR® Green QPCR Master Mix (Agilent, Santa Clara, USA). The total reaction volume of 25 µL included 12.5 µL Brilliant II SYBR® Green QPCR Master Mix, 1.0 µL 10 µM forward primer, 1.0 µL 10 µM reverse primer, 1.0 µL cDNA template and 9.5 µL nuclease-free PCR-grade water. The RT-PCR program was 95°C for 10 min; 40 cycles of 95°C for 30 s, 60°C for 60 s and 72°C for 30 s. The primers (Table S3) were designed by the software Primer-Premier 5.0 (Palo Alto, Canada). The relative value of the expression level of each gene was calculated by comparing the cycle thresholds (CTs) of the target genes with that of the housekeeping gene (18S rRNA gene) using the 2^−ΔΔCT^ method as previously described [26]. The results were represented by means ± S.D. of three biological replicates.

## Supporting Information

Table S1 Pathway description of TDFs by searching the KEGGPATHWAY database.
(DOC)

Table S2 The annotation of TDFs by BLASTX and GOs description.
(DOC)

Table S3 The primers of differentially expressed TDFs and six known genes for quantitative RT-PCR.
(DOC)
