# Supplementary material for: Metabolic Profiles and cDNA-AFLP Analysis of Salvia miltiorrhiza and Salvia castanea Diel f. tomentosa Stib
Source: PLoS One. 2012 Jan 30;7(1):e29678. doi: 10.1371/journal.pone.0029678 (PMC3268763; doi:10.1371/journal.pone.0029678)
Supplement: Table S1 — Pathway description of TDFs by searching the KEGGPATHWAY database. (DOC) [file pone.0029678.s001.doc]

| NO. | Seq. Name | Seq. Description | E-Value | GOs description | Enzyme Codes |
| --- | --- | --- | --- | --- | --- |
| 1 | O743 | glyceraldehyde-3-phosphate dehydrogenase c subunit | 4.63E-10 | F:glyceraldehyde-3-phosphate dehydrogenase (phosphorylating) activity; C:cytosol; F:copper ion binding; C:cell wall; P:seed development; P:response to sucrose stimulus; F:zinc ion binding; C:nucleolus; P:response to salt stress; P:defense response to bacterium; P:oxidation reduction; P:response to heat; F:NAD or NADH binding; P:glycolysis; P:gluconeogenesis; C:mitochondrial envelope; C:chloroplast; P:response to cadmium ion; P:response to hydrogen peroxide; C:plasma membrane; C:apoplast | EC:1.2.1.12 |
| 2 | O722 | glyceraldehyde-3-phosphate dehydrogenase | 2.62E-13 | F:glyceraldehyde-3-phosphate dehydrogenase (phosphorylating) activity; C:cytosol; F:copper ion binding; C:cell wall; P:seed development; P:response to sucrose stimulus; F:zinc ion binding; C:nucleolus; P:response to salt stress; P:defense response to bacterium; P:oxidation reduction; P:response to heat; F:NAD or NADH binding; P:glycolysis; P:gluconeogenesis; C:mitochondrial envelope; C:chloroplast; P:response to cadmium ion; P:response to hydrogen peroxide; C:plasma membrane; C:apoplast | EC:1.2.1.12 |
| 3 | D822 | catalase | 5.61E-08 | P:photoperiodism; P:transport; P:hydrogen peroxide catabolic process; C:mitochondrion; P:cell death; F:cobalt ion binding; P:response to cold; P:cell redox homeostasis; P:oxidation reduction; F:catalase activity; C:plant-type vacuole membrane; F:protein binding; F:heme binding; P:cellular response to sulfate starvation; P:cellular response to nitrogen starvation; C:cytosolic ribosome; P:cellular response to phosphate starvation; C:glyoxysome; C:plasma membrane; C:stromule | EC:1.11.1.6 |
| 4 | C841 | catalase | 5.50E-19 | P:photoperiodism; P:transport; P:hydrogen peroxide catabolic process; C:peroxisome; C:mitochondrion; P:cell death; F:cobalt ion binding; P:response to cold; P:cell redox homeostasis; P:oxidation reduction; F:catalase activity; C:plant-type vacuole membrane; F:protein binding; F:heme binding; P:cellular response to sulfate starvation; P:cellular response to nitrogen starvation; C:cytosolic ribosome; P:cellular response to phosphate starvation; C:plasma membrane; C:stromule | EC:1.11.1.6 |
| 5 | M611 | protein kinase-coding resistance protein | 5.25E-38 | C:cytosol; F:beta-amylase activity; P:response to water deprivation; C:integral to membrane; P:regulation of meristem growth; P:gametophyte development; P:oxidation reduction; F:2-alkenal reductase activity; F:receptor activity; P:starch catabolic process; P:transmembrane receptor protein tyrosine kinase signaling pathway; C:cytoplasmic membrane-bounded vesicle; F:ATP binding; F:protein binding; P:regulation of meristem structural organization; P:microsporocyte differentiation; F:MAP kinase kinase kinase activity; C:chloroplast; C:nucleus; P:protein amino acid phosphorylation | EC:3.2.1.2; EC:1.3.1.74; EC:2.7.11.25 |
| 6 | O741 | atp-citrate lyase a-2 | 2.63E-34 | P:carotenoid biosynthetic process; P:starch biosynthetic process; F:ATP citrate synthase activity; P:positive regulation of flower development; P:aging; P:anthocyanin accumulation in tissues in response to UV light; P:chlorophyll biosynthetic process; C:cytosol; P:positive regulation of cell size; P:regulation of embryonic development; C:citrate lyase complex; F:lyase activity; P:leaf development; P:wax biosynthetic process; P:acetyl-CoA biosynthetic process; F:ATP binding; F:succinate-CoA ligase (ADP-forming) activity | EC:2.3.3.8; EC:6.2.1.5 |
| 7 | M512 | ztl | 4.39E-29 | C:cytosol; P:response to red light; P:regulation of transcription, DNA-dependent; C:SCF ubiquitin ligase complex; P:flower development; P:entrainment of circadian clock by photoperiod; F:ubiquitin-protein ligase activity; F:protein binding; F:scavenger receptor activity; P:signal transduction; F:blue light photoreceptor activity; P:SCF-dependent proteasomal ubiquitin-dependent protein catabolic process; C:membrane; C:nucleus | EC:6.3.2.19 |
| 8 | I112 | heat shock protein 70 | 2.29E-38 | C:cytosol; C:cell wall; P:auxin biosynthetic process; P:protein folding; P:oxidation reduction; F:2-alkenal reductase activity; P:response to heat; F:ATP binding; C:chloroplast; C:vacuole; P:response to cadmium ion; C:cytosolic ribosome; C:plasma membrane; C:apoplast | EC:1.3.1.74 |
| 9 | J823 | transport inhibitor response 1 | 4.86E-20 | P:ubiquitin-dependent protein catabolic process; C:SCF ubiquitin ligase complex; F:inositol hexakisphosphate binding; F:auxin binding; P:lateral root formation; F:ubiquitin-protein ligase activity; F:protein binding; P:stamen development; P:response to molecule of bacterial origin; P:pollen maturation; P:auxin mediated signaling pathway; P:cellular response to phosphate starvation | EC:6.3.2.19 |
| 10 | B223 | citrate synthase | 1.37E-14 | F:zinc ion binding; F:lyase activity; F:acyltransferase activity; F:citrate (Si)-synthase activity; F:ATP citrate synthase activity; P:tricarboxylic acid cycle; P:cellular carbohydrate metabolic process; F:ATP binding; C:mitochondrial matrix; C:chloroplast; P:response to cadmium ion | EC:2.3.3.1; EC:2.3.3.8 |
| 11 | N811 | phosphoenolpyruvate carboxylase | 4.38E-05 | F:phosphoenolpyruvate carboxylase activity; F:2-alkenal reductase activity; P:photosynthesis; P:carbon fixation; C:cytoplasm; P:tricarboxylic acid cycle; P:oxidation reduction; F:lyase activity; F:oxidoreductase activity; F:catalytic activity |  |
| 12 | N621 | vacuolar h+-translocating inorganic pyrophosphatase | 4.98E-17 | F:binding; P:oxidation reduction; P:tyrosine biosynthetic process; F:2-alkenal reductase activity; F:prephenate dehydrogenase (NADP+) activity; C:cytoplasmic membrane-bounded vesicle; F:inorganic diphosphatase activity; P:proton transport; F:hydrogen-translocating pyrophosphatase activity; C:membrane | EC:1.3.1.74; EC:1.3.1.13; EC:3.6.1.1 |
| 13 | A121 | ubiquitin | 3.42E-13 | P:ubiquitin-dependent protein catabolic process; P:response to UV-B; C:nucleolus; P:response to salicylic acid stimulus; P:protein ubiquitination; F:protein binding; P:aging; C:vacuole; C:cytosolic large ribosomal subunit |  |
| 14 | E844 | hd domain class transcription factor | 2.01E-05 | P:regulation of transcription, DNA-dependent; P:transcription; F:sequence-specific DNA binding; P:regulation of transcription; F:transcription regulator activity; F:DNA binding; F:transcription factor activity; C:nucleus |  |
| 15 | P113 | 2-dehydro-3-deoxyphosphoheptonate aldolase 3-deoxy-d-arabino-heptulosonate 7-phosphate synthetase | 7.46E-08 | P:response to wounding; P:response to bacterium; F:protein binding; P:chorismate biosynthetic process; F:3-deoxy-7-phosphoheptulonate synthase activity; P:aromatic amino acid family biosynthetic process; F:lyase activity; C:chloroplast | EC:2.5.1.54 |
| 16 | K313 | pectin methylesterase | 2.03E-26 | F:pectinesterase activity; P:cell wall modification; C:plant-type cell wall; C:cytoplasm; C:apoplast; F:aspartyl esterase activity; P:response to nematode; F:enzyme inhibitor activity | EC:3.1.1.11 |
| 17 | D335 | atp synthase subunit 1 | 8.44E-12 | C:proton-transporting ATP synthase complex, catalytic core F(1); P:plasma membrane ATP synthesis coupled proton transport; P:auxin biosynthetic process; F:hydrogen ion transporting ATP synthase activity, rotational mechanism; C:mitochondrion; F:ATP binding; F:proton-transporting ATPase activity, rotational mechanism | EC:3.6.3.14 |
| 18 | D311 | atp synthase subunit 1 | 2.89E-12 | C:proton-transporting ATP synthase complex, catalytic core F(1); P:plasma membrane ATP synthesis coupled proton transport; P:auxin biosynthetic process; F:hydrogen ion transporting ATP synthase activity, rotational mechanism; C:mitochondrion; F:ATP binding; F:proton-transporting ATPase activity, rotational mechanism | EC:3.6.3.14 |
| 19 | E841 | eukaryotic initiation factor eif4 | 5.27E-14 | C:cytoplasm; P:translational initiation; F:RNA binding; F:protein binding; F:translation initiation factor activity; P:RNA metabolic process; C:nucleus |  |
| 20 | F225 | protein | 4.97E-17 | C:integral to endoplasmic reticulum membrane; P:protein folding; C:cytoplasmic membrane-bounded vesicle; P:response to stress; F:heat shock protein binding; F:unfolded protein binding; C:mitochondrion |  |
| 21 | P137 | 2-dehydro-3-deoxyphosphoheptonate aldolase 3-deoxy-d-arabino-heptulosonate 7-phosphate synthetase | 1.07E-06 | C:plastid; C:chloroplast; F:transferase activity; F:3-deoxy-7-phosphoheptulonate synthase activity; P:cellular amino acid biosynthetic process; P:aromatic amino acid family biosynthetic process |  |
| 22 | H321 | hexose transporter | 6.03E-10 | P:transmembrane transport; P:oxidation reduction; P:carbohydrate transport; C:integral to membrane; F:high-affinity hydrogen:glucose symporter activity; F:2-alkenal reductase activity | EC:1.3.1.74 |
| 23 | O843 | kinase family protein | 4.60E-10 | P:auxin biosynthetic process; P:protein amino acid phosphorylation; F:ATP binding; P:signal transduction; F:protein serine/threonine kinase activity; C:mitochondrion | EC:2.7.11.0 |
| 24 | M725 | gibberellin receptor | 4.90E-12 | P:positive regulation of gibberellic acid mediated signaling pathway; P:floral organ morphogenesis; F:receptor activity; F:carboxylesterase activity; F:protein binding; P:raffinose family oligosaccharide biosynthetic process | EC:3.1.1.0; EC:3.1.1.1 |
| 25 | G513 | kinase-like protein | 1.64E-15 | P:auxin biosynthetic process; P:protein amino acid phosphorylation; F:non-membrane spanning protein tyrosine kinase activity; C:plasma membrane; F:ATP binding; F:protein serine/threonine kinase activity | EC:2.7.10.2; EC:2.7.11.0 |
| 26 | O742 | 60s ribosomal protein l21 | 5.53E-16 | C:nucleolus; F:structural constituent of ribosome; C:cytosolic large ribosomal subunit; C:chloroplast; P:translation; C:mitochondrion | EC:3.6.5.3 |
| 27 | D812 | udp-glucose 6- | 5.48E-19 | C:cytosol; F:UDP-glucose 6-dehydrogenase activity; P:oxidation reduction; C:cell wall; F:NAD or NADH binding; C:nucleus | EC:1.1.1.22 |
| 28 | A721 | actin | 8.92E-22 | C:cytoplasm; P:auxin biosynthetic process; C:cytoskeleton; F:protein binding; F:ATP binding; C:nucleus |  |
| 29 | D221 | pyruvate kinase | 1.19E-34 | C:cytoplasm; F:pyruvate kinase activity; F:potassium ion binding; F:ATP binding; F:magnesium ion binding; P:glycolysis | EC:2.7.1.40 |
| 30 | M511 | nbs-coding resistance gene analog | 4.41E-05 | F:ATP binding; P:apoptosis; P:defense response; F:nucleotide binding; F:transferase activity, transferring acyl groups other than amino-acyl groups |  |
| 31 | O711 | protein | 2.13E-07 | F:oxidoreductase activity; F:protein kinase activity; P:cellular metabolic process; C:membrane; F:nucleotide binding |  |
| 32 | M812 | betaine aldehyde dehydrogenase | 6.92E-11 | P:oxidation reduction; F:betaine-aldehyde dehydrogenase activity; F:3-chloroallyl aldehyde dehydrogenase activity; C:chloroplast; F:aminobutyraldehyde dehydrogenase activity | EC:1.2.1.8; EC:1.2.1.19 |
| 33 | D515 | protein | 9.97E-13 | P:response to auxin stimulus; P:vegetative to reproductive phase transition of meristem; F:protein binding; C:plasma membrane; P:xylem and phloem pattern formation |  |
| 34 | D112 | cytochrome c oxidase subunit 2 | 8.99E-14 | F:transition metal ion binding; F:oxidoreductase activity; C:membrane part; C:mitochondrion; P:electron transport chain |  |
| 35 | M133 | vacuolar proton-inorganic pyrophosphatase | 6.84E-14 | F:hydrogen-translocating pyrophosphatase activity; C:vacuolar membrane; P:proton transport; F:inorganic diphosphatase activity; C:integral to membrane | EC:3.6.1.1 |
| 36 | G711 | coatomer gamma | 1.36E-14 | F:protein binding; P:vesicle-mediated transport; P:intracellular protein transport; C:COPI vesicle coat; F:structural molecule activity |  |
| 37 | N741 | pyruvate decarboxylase | 1.93E-16 | F:thiamin pyrophosphate binding; P:response to hypoxia; F:magnesium ion binding; F:transferase activity; F:pyruvate decarboxylase activity | EC:4.1.1.1 |
| 38 | E213 | sumo activating enzyme 2 | 4.52E-18 | P:protein sumoylation; P:embryonic development ending in seed dormancy; F:ATP binding; C:cytosol; F:SUMO activating enzyme activity |  |
| 39 | N742 | pyruvate decarboxylase | 2.44E-19 | F:thiamin pyrophosphate binding; P:response to hypoxia; F:magnesium ion binding; F:transferase activity; F:pyruvate decarboxylase activity | EC:4.1.1.1 |
| 40 | E226 | sumo activating enzyme 2 | 6.27E-20 | P:protein sumoylation; P:embryonic development ending in seed dormancy; F:ATP binding; C:cytosol; F:SUMO activating enzyme activity |  |
| 41 | A542 | actin- expressed | 1.77E-22 | C:cytoplasm; P:auxin biosynthetic process; C:cytoskeleton; F:protein binding; F:ATP binding |  |
| 42 | M442 | pyruvate decarboxylase | 8.33E-28 | F:thiamin pyrophosphate binding; C:plastid; F:magnesium ion binding; F:transferase activity; F:pyruvate decarboxylase activity | EC:4.1.1.1 |
| 43 | M443 | pyruvate decarboxylase | 6.75E-30 | F:thiamin pyrophosphate binding; C:plastid; F:magnesium ion binding; F:transferase activity; F:pyruvate decarboxylase activity | EC:4.1.1.1 |
| 44 | P711 | atp binding | 3.22E-08 | P:lipid transport; F:lipid transporter activity; C:cytosol; C:nucleus |  |
| 45 | H216 | homeobox-leucine zipper protein | 9.39E-11 | F:sequence-specific DNA binding; F:transcription factor activity; C:nucleus; P:regulation of transcription, DNA-dependent |  |
| 46 | D336 | ubiquitin-conjugating enzyme e2 | 4.49E-13 | P:ubiquitin-dependent protein catabolic process; F:ubiquitin-protein ligase activity; P:regulation of protein metabolic process; P:post-translational protein modification | EC:6.3.2.19 |
| 47 | G843 | dihydroflavonol reductase | 4.61E-18 | C:cytoplasm; F:UDP-glucuronate decarboxylase activity; P:nucleotide-sugar biosynthetic process; F:NAD or NADH binding | EC:4.1.1.35 |
| 48 | D841 | protein | 2.30E-22 | F:NADP or NADPH binding; P:oxidation reduction; P:pentose-phosphate shunt; F:phosphogluconate dehydrogenase (decarboxylating) activity | EC:1.1.1.44 |
| 49 | P541 | protein | 1.36E-22 | C:cytoplasm; P:regulation of transcription; F:transcription factor activity; F:zinc ion binding |  |
| 50 | G841 | lipoxygenase | 4.19E-24 | P:oxylipin biosynthetic process; F:iron ion binding; P:oxidation reduction; F:lipoxygenase activity | EC:1.13.11.12 |
| 51 | P321 | cytochrome p450 like_tbp | 6.11E-07 | P:oxidation reduction; F:oxidoreductase activity; F:aromatase activity |  |
| 52 | D441 | s-receptor kinase-like | 4.22E-08 | F:protein kinase activity; P:cellular metabolic process; F:nucleotide binding |  |
| 53 | G741 | jasmonate zim-domain protein 3 | 1.45E-08 | P:biological_process; F:protein binding; C:cellular_component |  |
| 54 | C441 | protein | 2.43E-11 | C:exocyst; F:protein binding; P:vesicle docking during exocytosis |  |
| 55 | M722 | phosphatidate cytidylyltransferase family protein | 3.71E-12 | C:membrane; C:cytoplasmic membrane-bounded vesicle; F:transferase activity |  |
| 56 | H215 | glycine-rich rna-binding protein | 6.49E-17 | C:nucleus; F:nucleotide binding; F:RNA binding |  |
| 57 | K511 | transferring glycosyl | 3.84E-17 | C:obs,3-beta-glucan synthase complex; F:obs,3-beta-glucan synthase activity; P:obs,3-beta-glucan biosynthetic process | EC:2.4.1.34 |
| 58 | K611 | glycoside hydrolase family 48 | 2.24E-17 | F:hydrolase activity, hydrolyzing O-glycosyl compounds; P:carbohydrate metabolic process; F:polysaccharide binding | EC:3.2.1.0 |
| 59 | O641 | cinnamyl alcohol dehydrogenase-like protein | 2.36E-35 | F:zinc ion binding; P:oxidation reduction; F:mannitol dehydrogenase activity | EC:1.1.1.255 |
| 60 | C121 | conserved protein | 3.33E-05 | C:plastid; C:chloroplast |  |
| 61 | P441 | ein3-like protein | 3.68E-07 | C:nucleus; F:transcription regulator activity |  |
| 62 | L114 | pre-mrna-splicing factor cwc-22 | 1.26E-07 | F:protein binding; P:RNA metabolic process |  |
| 63 | D512 | transcription factor, putative (Ricinus communis] | 2.09E-10 | C:intracellular; F:DNA binding |  |
| 64 | G842 | patatin b2 | 1.18E-10 | P:lipid catabolic process; F:hydrolase activity |  |
| 65 | N721 | pyruvate decarboxylase | 4.06E-11 | F:binding; F:lyase activity |  |
| 66 | K234 | gpi-anchored protein | 9.91E-13 | C:anchored to membrane; C:cytoplasmic membrane-bounded vesicle |  |
| 67 | M613 | bzip protein | 4.65E-15 | F:DNA binding; C:plasma membrane |  |
| 68 | B323 | cytochrome p450 like_tbp | 1.46E-16 | P:oxidation reduction; F:aromatase activity |  |
| 69 | P324 | motor axon guidance family member (max-2) | 7.38E-21 | P:oxidation reduction; F:aromatase activity |  |
| 70 | F812 | lipoprotein | 4.60E-26 | F:hydrolase activity; C:plastid |  |
| 71 | G237 | glutathione s-transferase | 2.69E-26 | P:response to stress; F:glutathione transferase activity | EC:2.5.1.18 |
| 72 | N441 | lectin | 9.36E-08 | F:binding |  |
| 73 | P112 | metallothionein-like protein | 3.32E-08 | F:metal ion binding |  |
| 74 | F411 | 21 kda protein | 1.71E-12 | F:pectinesterase inhibitor activity |  |
| 75 | J822 | cell wall-associated hydrolase | 8.77E-13 | F:hydrolase activity |  |
| 76 | L722 | cell wall-associated hydrolase | 6.47E-20 | F:hydrolase activity |  |
| 77 | M144 | protein | 6.53E-25 | C:mitochondrion |  |
| 78 | C126 | protein | 9.91E-29 | C:mitochondrion |  |
