# Supplementary material for: Metabolic Profiles and cDNA-AFLP Analysis of Salvia miltiorrhiza and Salvia castanea Diel f. tomentosa Stib
Source: PLoS One. 2012 Jan 30;7(1):e29678. doi: 10.1371/journal.pone.0029678 (PMC3268763; doi:10.1371/journal.pone.0029678)
Supplement: Table S2 — The annotation of TDFs by BLASTX and GOs description. (DOC) [file pone.0029678.s002.doc]

| Seq. Name | Pathway | Enzyme | Enzyme codes |
| --- | --- | --- | --- |
| B223 | Biosynthesis of alkaloids | citrate (Si)-synthase | EC:2.3.3.1 |
|  | Biosynthesis of phenylpropanoids |  |  |
|  | Biosynthesis of plant hormones |  |  |
|  | Biosynthesis of terpenoids and steroids |  |  |
|  | Citrate cycle (TCA cycle) |  |  |
|  | Glyoxylate and dicarboxylate metabolism |  |  |
| B223, O741 | Biosynthesis of alkaloids | ATP citrate synthase | EC:2.3.3.8 |
|  | Biosynthesis of phenylpropanoids |  |  |
|  | Biosynthesis of plant hormones |  |  |
|  | Biosynthesis of terpenoids and steroids |  |  |
|  | Carbon fixation pathways in prokaryotes |  |  |
|  | Citrate cycle (TCA cycle) |  |  |
| C841, D822 | Methane metabolism | catalase | EC:1.11.1.6 |
|  | Tryptophan metabolism |  |  |
| D221 | Biosynthesis of alkaloids | pyruvate kinase | EC:2.7.1.40 |
|  | Biosynthesis of phenylpropanoids |  |  |
|  | Biosynthesis of plant hormones |  |  |
|  | Biosynthesis of terpenoids and steroids |  |  |
|  | Carbon fixation in photosynthetic organisms |  |  |
|  | Glycolysis / Gluconeogenesis |  |  |
|  | Purine metabolism |  |  |
|  | Pyruvate metabolism |  |  |
| D311, D335 | Methane metabolism | H+-transporting two-sector ATPase | EC:3.6.3.14 |
|  | Oxidative phosphorylation |  |  |
|  | Photosynthesis |  |  |
| D812 | Amino sugar and nucleotide sugar metabolism | UDP-glucose 6-dehydrogenase | EC:1.1.1.22 |
|  | Ascorbate and aldarate metabolism |  |  |
|  | Biosynthesis of secondary metabolites |  |  |
|  | Pentose and glucuronate interconversions |  |  |
|  | Starch and sucrose metabolism |  |  |
| D841 | Biosynthesis of alkaloids | phosphogluconate dehydrogenase (decarboxylating) | EC:1.1.1.44 |
|  | Biosynthesis of plant hormones |  |  |
|  | Biosynthesis of secondary metabolites |  |  |
|  | Glutathione metabolism |  |  |
|  | Pentose phosphate pathway |  |  |
| G237 | Glutathione metabolism | glutathione transferase | EC:2.5.1.18 |
| G841 | alpha-Linolenic acid metabolism | lipoxygenase | EC:1.13.11.12 |
|  | Biosynthesis of plant hormones |  |  |
|  | Linoleic acid metabolism |  |  |
| G843 | Amino sugar and nucleotide sugar metabolism | UDP-glucuronate decarboxylase | EC:4.1.1.35 |
|  | Starch and sucrose metabolism |  |  |
| K313 | Pentose and glucuronate interconversions | pectinesterase | EC:3.1.1.11 |
|  | Starch and sucrose metabolism |  |  |
| K511 | Starch and sucrose metabolism | 1,3-beta-glucan synthase | EC:2.4.1.34 |
| M133, N621 | Oxidative phosphorylation | inorganic diphosphatase ec:3.6.1.10 endopolyphosphatase | EC:3.6.1.1 |
| M611 | Starch and sucrose metabolism | beta-amylase | EC:3.2.1.2 |
| M725 | Biosynthesis of secondary metabolites | carboxylesterase | EC:3.1.1.1 |
|  | Tropane, piperidine and pyridine alkaloid biosynthesis |  |  |
| M812 | Arginine and proline metabolism | aminobutyraldehyde dehydrogenase | EC:1.2.1.19 |
|  | beta-Alanine metabolism |  |  |
|  | Glycine, serine and threonine metabolism |  |  |
| N621 | Biosynthesis of secondary metabolites | prephenate dehydrogenase (NADP+) | EC:1.3.1.13 |
|  | Phenylalanine, tyrosine and tryptophan biosynthesis |  |  |
| N741, N742, M442 | Biosynthesis of secondary metabolites | pyruvate decarboxylase | EC:4.1.1.1 |
|  | Glycolysis / Gluconeogenesis |  |  |
| O741 | Biosynthesis of alkaloids | succinate---CoA ligase (ADP-forming) | EC:6.2.1.5 |
|  | Biosynthesis of phenylpropanoids |  |  |
|  | Biosynthesis of plant hormones |  |  |
|  | Biosynthesis of terpenoids and steroids |  |  |
|  | C5-Branched dibasic acid metabolism |  |  |
|  | Carbon fixation pathways in prokaryotes |  |  |
|  | Citrate cycle (TCA cycle) |  |  |
|  | Propanoate metabolism |  |  |
| O743, O722 | Biosynthesis of alkaloids | glyceraldehyde-3-phosphate dehydrogenase (phosphorylating) | EC:1.2.1.12 |
|  | Biosynthesis of phenylpropanoids |  |  |
|  | Biosynthesis of plant hormones |  |  |
|  | Glycolysis / Gluconeogenesis |  |  |
|  | Biosynthesis of terpenoids and steroids |  |  |
| P113 | Biosynthesis of alkaloids | 3-deoxy-7-phosphoheptulonate synthase | EC:2.5.1.54 |
|  | Biosynthesis of phenylpropanoids |  |  |
|  | Biosynthesis of plant hormones |  |  |
|  | Phenylalanine, tyrosine and tryptophan biosynthesis |  |  |
