# Supplementary material for: Metabolic Profiles and cDNA-AFLP Analysis of Salvia miltiorrhiza and Salvia castanea Diel f. tomentosa Stib
Source: PLoS One. 2012 Jan 30;7(1):e29678. doi: 10.1371/journal.pone.0029678 (PMC3268763; doi:10.1371/journal.pone.0029678)
Supplement: Table S3 — The primers of differentially expressed TDFs and six known genes for quantitative RT-PCR. (DOC) [file pone.0029678.s003.doc]

| Gene | Forward Primer (5´→3´) | Reverse Primer (5´→3´) |
| --- | --- | --- |
| *HMGR* | GCAACATCGTCTCCGCCGTCTACA | GATGGTGGCCAGCAGCCTGGAGTT |
| *DXR* | CATGCGTTTGCCTATTCTGTAC | ACTAAGAACTCCGGTCATGGTG |
| *KS* | CTTCCCAAGACAATGCAAAGAT | ATTTCCCTCTCACATTATTAGC |
| *PAL* | ACCTACCTCGTCGCCCTATGC | CCACGCGGATCAAGTCCTTCT |
| *TAT* | TTCAACGGCTACGCTCCAACT | AAACGGACAATGCTATCTCAAT |
| *RAS* | CACAGTTTCCGGTGCCCTAAT | CGTGATGGCGACGAACAAGC |
| A148 | TTGGCATATCCCTCACAAGAG | GTTCGTCGAAAGCGCAAGA |
| A346 | TCGGGGCTAGTTGATTCGGCAG | AACAGCAGGACGGTGGTCATGGA |
| A1410 | GATTCTTTTGGCATATCCCTCAC | GTTCGTCGAAAGCGCAAGAC |
| C841 | TGGACGTGACGCTGACGTGG | GGCCAGCTGCTCATTCTCCG |
| E844 | ACCTCAAATCCACCTACGACG | CAACCGATCATTCAGCTCCTG |
| G741 | TCATCACCCTCTCCTTACGCTTC | ATTTCATCAGCTGTTCCACAGGC |
| G841 | GAAATACGAGAAAAGGGACATGG | TAGTGGTGCAGGATTCTATCAGC |
| G843 | TCTATGCGAGAAGCTGATGGAG | TGTTGAGACGGTGGAACTGGAT |
| I842 | CCATTTTCGGGGCTAGTTGATTCGG | AGACAGCAGGACGGTGGTCATGGAA |
| L341 | TAGATTAGTCAAGCGCACTCACTCT | AGCCGCTGTTCCCATTTCCT |
| M442 | GCTTCCACCAACTCATCTTCC | GATGGCCCCTACAATGTGATC |
| N441 | GAGCCGAAATTCCGAGGTTGG | CCGATGGCAAAACCGCTGAC |
| O641 | ATCGTGCGTATCCCCGACAACA | ATGGGGCTGTAGGTGGTGATGC |
| O741 | TTGTCTTCTCATCAAGTCCGTG | TTGACGATACCGCTGCTTTC |
| O743 | GCACTTTCACATCGTTATGCTTC | GATCCATTCATCACCCACGAG |
| P541 | TCATTCAAAGCCTGTTGCTGG | AAGTCGCCTCCGATCCTCACT |
| *18s rRNA* | CCAGGTCCAGACATAGTAAG | GTACAAAGGGCAGGGACGTA |
